# Cerebrospinal fluid metabolomics reveals predictive biomarkers of nusinersen therapy efficacy in type II and type III spinal muscular atrophy patients

**DOI:** 10.1007/s10072-025-08267-8

**Published:** 2025-06-12

**Authors:** Dan Li, Na Sun, Hongbo Wu, Xueying Wang, Yongjing Shi, Lin Yang, Shaoping Huang, Kun Zhang, Changhong Yang

**Affiliations:** 1 Department of Pediatrics, the Second Affiliated Hospital of Xi’an Jiaotong University, No.157 Xiwu road, Xi’an, 710004 Shaanxi province China; 2 Department of Pediatrics, Hanzhong Central Hospital, Hanzhong, Shaanxi China

**Keywords:** Spinal muscular atrophy (SMA), Nusinersen, Cerebrospinal fluid (CSF), Metabolomics, Liquid chromatography-tandem mass spectrometry (LC-MS/MS)

## Abstract

**Background and purpose:**

This study investigated the predictive value of clinical characteristics and cerebrospinal fluid (CSF) metabolites for nusinersen efficacy in children with spinal muscular atrophy (SMA).

**Methods:**

In this study, clinical data and CSF samples were collected. We used liquid chromatography-tandem mass spectrometry to analyze CSF metabolites from 42 patients with type II and type III SMA.

**Results:**

Although clinical indicators, such as age at treatment initiation and disease duration, did not predict the clinical efficacy of nusinersen, we identified 47 differentially expressed metabolites between effective- and ineffective-treatment patients with type II disease and 109 metabolites in patients with type III disease. Additionally, KEGG-enriched pathway analysis revealed differences in several pathways between the effective- and ineffective-treatment groups for both types II and III. N-myristoyl arginine and 1,1,1,2,2,2-Pentafluoro-7-phenylheptan-3-one were negatively associated with Hammersmith Functional Motor Scale Expanded changes in patients with type III and type III SMA. Furthermore, multivariate receiver operating characteristic curve analysis indicated that differential metabolites have some accuracy in predicting SMA treatment efficacy.

**Conclusion:**

This study identified CSF metabolites that are predictive of nusinersen efficacy. The results of this study may guide the development of adjunctive therapies for improving the efficacy of nusinersen.

**Supplementary Information:**

The online version contains supplementary material available at 10.1007/s10072-025-08267-8.

## Introduction

Spinal muscular atrophy (SMA) is a hereditary neuromuscular disorder, mainly divided into 5q-SMA and non-5q-SMA, which differ in clinical features, genetic mechanisms, and treatment status. 5q-SMA is caused by biallelic mutations in the Survival Motor Neuron 1 gene (SMN1) located on chromosome 5q13, and has genetic homogeneity. The incidence rate is 1/10,000 - 20,000 in live births, and the carrier frequency is 1/40 - 70. The main manifestations are symmetrical proximal muscle weakness, with the lower limbs more severely affected than the upper limbs. Respiratory failure is a serious complication. Diagnosis relies on techniques such as MLPA and PCR. Non-5q-SMA has high genetic heterogeneity, and a relatively low incidence rate, and the diagnosis depends on DNA analysis techniques such as whole exome sequencing and whole genome sequencing [1]. 5q SMA is classically classified according to the age at onset and the maximum motor milestones achieved. Children with type I SMA, who account for approximately 60% of all patients with SMA, develop SMA within the first 6 months of life, present with flaccid limbs, and never achieve the ability to sit independently. Approximately 30% of the patients are diagnosed with type II SMA. These patients develop muscle weakness in late infancy and gain the ability to sit independently, but do not walk. Patients who can walk are diagnosed with type III SMA, accounting for approximately 10% of all patients [2]. In addition, patients are classified based on functional status: walkers (able to walk at least five steps without assistance), sitters (able to sit without assistance nor head support for more than 10 s), and non-sitters [3].

Nusinersen is an antisense oligonucleotide designed to modify *SMN2* gene splicing to increase exon 7 expression and thereby increase full-length *SMN* protein expression [4]. It was approved by the US Food and Drug Administration in late December 2016 and by the Chinese Drug Administration in February 2019. However, due to its high price, it cannot be accepted by most Chinese SMA families. From January 1, 2022, the National Health Insurance Administration in China will reimburse most of the costs of nusinersen treatment for all clinical types of pediatric 5qSMA on a case-by-case basis. At this point, most SMA patients and their families begin to choose treatment. Although the proportion of patients with SMA type I was the highest in theory, due to its high mortality rate, the proportion of patients with SMA type I was very low in reality, which could not be statistically analyzed. Therefore, this part of the data was removed from the data analysis of this paper.

The clinical heterogeneity of SMA complicates the prediction of the efficacy of nusinersen [5]. Patients treated earlier were found to perform better in terms of functional improvement [6]，while other investigators have suggested that there was no significant difference in the treatment effect between adults and children [7]. Moreover, the correlation between SMA phenotype and *SMN2* copy number is not absolute, and genetic modifiers other than *SMN2* gene may affect the disease progression [8]. Therefore, *SMN2* copy number could not accurately predict the efficacy of nusinersen [9]. Combinedly, all of these factors make it difficult to predict the efficacy of nusinersen.

Metabolomics plays a key role in the research of neurodegenerative diseases, which can reveal the mechanism of disease, find biomarkers and evaluate the therapeutic effect [10]. Metabolomics can simultaneously detect a large number of metabolites in biological samples, covering a variety of types, and can comprehensively present the metabolic picture of an organism at a particular time. This comprehensive detection approach helps to discover metabolic pathways and biomarkers that have not been paid attention to before, and provides rich data support for in-depth understanding of disease mechanisms [11]. Metabolomics can also identify specific metabolites associated with neurodegenerative diseases, which can serve as potential biomarkers for early diagnosis and disease monitoring. For instance, in the study of SMA, metabolomic analysis of patients’ cerebrospinal fluid (CSF) has identified certain metabolites related to the disease state and severity of SMA, such as specific lipids and amino acids. These metabolites may become biomarkers for diagnosing SMA and assessing disease progression [12].

CSF is a useful biofluid for biomarker screening in SMA cases because it is in direct contact with the affected tissues (lower brainstem and spinal cord). Furthermore, samples can be collected without further invasive surgery before nusinersen is injected into a patient's CSF.

A few studies have been conducted on CSF metabolomics in patients with SMA. Errico et al. reported altered CSF metabolites after nusinersen treatment, which was associated with disease severity. However, the researchers did not prospectively investigate the differences in the CSF metabolites between the effective- and ineffective-treatment groups. We conducted a prospective case-cohort study to investigate the predictive value of clinical characteristics or cerebrospinal fluid metabolites in children with SMA for nusinersen efficacy.

## Materials and Methods

### Study design, patients, and ethics

Forty-five children with SMA type II and III were initially enrolled in this study. All patients were diagnosed with 5qSMA at the Second Affiliated Hospital of Xi'an Jiaotong University between August 2022 and December 2023 and received nusinersen treatment. At first, two senior pediatricians simultaneously diagnosed the patient. SMA diagnosis was confirmed if the two diagnoses were in agreement and assessed by a third experienced doctor if the diagnoses were not in agreement. The diagnosis was verified using Genetic testing (MLPA and nested PCR). Forty-two children were included in the study. Three were excluded: two did not complete nusinersen treatment as planned because of pneumonia and one was not assessed with the Hammersmith Functional Motor Scale Expanded (HFMSE) due to a radial fracture. Only patients who were assessed using at least two HFMSE at 14-month intervals were enrolled in this study. We excluded patients who had been treated with other therapies, such as risdiplam and salbutamol, before or concurrently with nusinersen. We also excluded patients whose blood was mixed during CSF collection. Patients were excluded from the analysis if their HFMSE scores were considered unreliable by the examiners because of interfering factors, such as temporary pain. This clinical trial was registered in China under MR-61-22-007220 on twenty-sixth July 2022. https://www.medicalresearch.org.cn/.

**Fig. 1:**
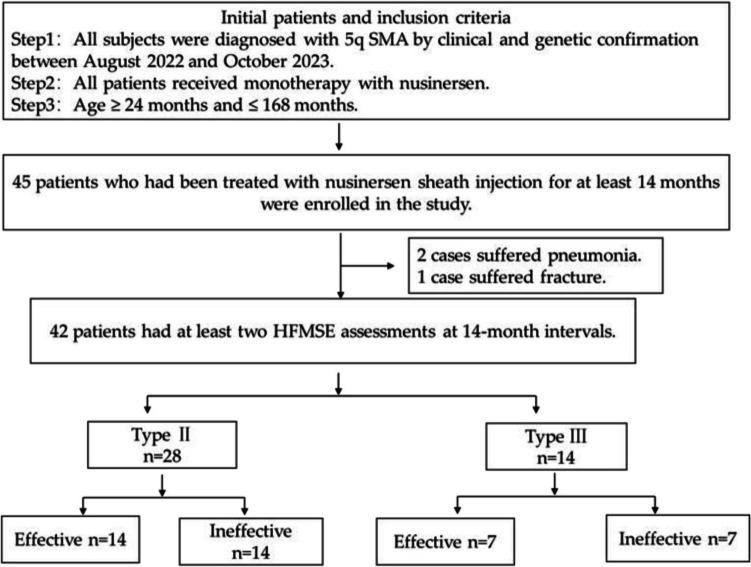
Clinical trial flow chart

Clinical data, including age, age at onset, age at genetic diagnosis, disease duration, HFMSE scores, *SMN2* copy number, spinal radiographs, and CSF metabolomics, were collected from 42 patients with SMA at baseline. The observational endpoint was set at month 14 of nusinersen treatment and the HFMSE was reevaluated. The Clinical trial flow chart was seen in Fig. 1. Clinical trial flow chart.

All children's guardians and children aged ≥8 years gave their informed consent for inclusion before participating in the study. The study was conducted in accordance with the Declaration of Helsinki and the protocol was approved by the Ethics Committee of the Second Affiliated Hospital of Xi’an Jiaotong University (2022 Ethical Approval Study No. 038). This clinical trial was registered in China under MR-61-22-007220. https://www.medicalresearch.org.cn/.

### Nusinersen therapeutic procedures

A loading dose of 5 mL/dose was administered via intrathecal injection four times in the first 2 months. During the maintenance phase, 5 mL of nusinersen was administered once every 4 months.

### HFMSE score

This scale consists of 33 items assessing a child's ability to perform various activities. The total score ranged from 0 (failure in all activities) to 66 (completion of all activities). All items were tested without a back brace or orthosis.

Giorgia Coratti et al. [13] analyzed the natural history of type II and founded that the HFMSE score was stable at ± 2 in 68% and 55% of patients at 12 and 24 months of observation, respectively. Therefore, the fluctuation of HFMSE before and after treatment (±2 points) may be due to the natural stability of the disease, rather than the effect of nusinersen. So most researchers agreed that an improvement of ≥ 3 points from baseline in the final HFMSE score was considered clinically effective [13–16].

### CSF sample preparation and liquid chromatography-tandem mass spectrometry acquisition

CSF samples were centrifuged at 800 × *g* for 5 min immediately after collection and stored in freeze tubes (500 μL/tube)at −80 °C for backup. After defrosting, metabolites were extracted by mixing samples in a centrifuge tube at a ratio of 100 μL of CSF and 400 μL of 0.02 mg/mL internal standard solution (L-2-chlorophenylalanine).

CSF samples were processed using established procedures [17] including stirring, low-temperature sonication, −20 °C protein precipitation, centrifugation, nitrogen drying, re-dissolution, low-temperature sonication extraction, and centrifugation. The resulting supernatant was analyzed using liquid chromatography-tandem mass spectrometry (LC-MS/MS).

Quality control (QC) samples were prepared by pooling portions of samples. QC samples were handled and assayed in the same manner as the analytical samples. This ensured that a complete set of samples was represented. These were checked at intervals (every 5–15 samples) to maintain analytical consistency.

A Thermo UHPLC-Q Exactive system (Majorbio Biomedical Technology Ltd., Shanghai, China) was used for ultra-performance liquid chromatography/tandem mass spectrometry (UPLC-MS/MS) analysis. The ACQUITY HSS T3 column (100 mm × 2.1 mm i.d. 1.8 μm; Waters Corporation, MA, USA) was used in the system. The mobile phase consisted of two solvents: solvent A (0.1% formic acid in water and acetonitrile at a 95:5 ratio) and solvent B (0.1% formic acid in a mixture of acetonitrile, isopropanol, and water). The injection volume was 3 μL. The column temperature was maintained at 40 °C and the flow rate was 0.40 mL/min.

### Data processing

LC-MS data were preprocessed using Progenesis QI software (version 3.0) (Waters Corporation, MA, USA). Metabolite identification was performed using the Majorbio, Metlin (https://metlin.scripps.edu/), and HMDB (http://www.hmdb.ca/) databases. Data analysis was performed using the R Studio software package (version 4.2.2). Orthogonal partial least squares discriminant analysis (OPLS-DA) and 7-cycle interactive validation were conducted with the R package'ropls'(version 1.6.2) to assess model stability. The variable importance in projection (VIP) was measured using OPLS-DA. Student's *t*-test was used.

In this study, metabolites with *p* values < 0.05, VIP > 1, and FDR < 0.2 were considered statistically significant [17, 18]. Metabolic enrichment and pathway analysis via the KEGG database (http://www.genome.jp/kegg/) was used to map the differential metabolites between the two treatment groups to their respective biochemical pathways. R-software packages were used to plot heat maps, volcano maps, WGCNA, and receiver operating characteristic (ROC) curves.

### Statistical data analysis

Statistical data analysis was performed using SPSS software (version 24.0; IBM Corporation, Armonk, NY, USA). The data were subjected to the Shapiro–Wilk test for normality. Data that deviated from the normal distribution were presented as medians (interquartile range). Normally distributed data were presented as mean ± standard deviation. The Mann–Whitney rank test was used to compare two data groups that deviated from a normal distribution. Student’s *t*-test was used to compare two normally distributed groups.

## Results

### Clinical characters of participants

A total of 42 children diagnosed with SMA—28 with type II and 14 with type III SMA—were included in this study. There were 8 cases with 2 *SMN2* copies, 33 cases with 3 *SMN2* copies, and 1 case with 4 *SMN2* copies. Among the 42 patients, 21 cases were effective and 21 cases were ineffective. There were no significant differences in sex, scoliosis, genetic test results, HFMSE baseline, or CSF biochemistry between the patients who responded and those who did not respond to the therapy (*p* > 0.05). However, disease duration and age at initial treatment differed significantly between the groups divided by treatment efficacy (*p* < 0.05).

Considering that the indicators for predicting treatment efficacy may differ among clinical subtypes, the patients were divided into two subgroups, type II and type III, and the clinical data were compared according to efficacy. The age at treatment initiation and the duration of disease differed significantly between the two groups in type II patients; however, logistic regression analysis revealed no clinical predictive value (*p* > 0.05). Additionally, there were no significant differences in the clinical data between the two groups in type III patients (Table 1).

**Table 1:** Clinical characters of patients with SMA

Characteristics	Type II			Type III		
	Effective	Ineffective	*p* value	Effective	Ineffective	*p* value
n	14	14		7	7	
Age(months), median (IQR)	50 (31.5, 79)	93.5 (66, 108)	**0.032**^**a**^	96.29 ± 65.39	100.14 ± 44.48	0.899^b^
Age of onset(months), mean ± SD	11.79 ± 4.35	10.39 ± 5.39	0.470^b^	18 (18, 25)	24 (17.5, 33)	0.846^a^
Disease durings(months), median (IQR)	33.5 (19.5, 72.25)	77 (57.75, 100)	**0.034**^**a**^	59.57 ± 56.94	64.71± 51.25	0.862^b^
Gender, n (%)			0.131^c^			0.592^c^
Girls	9 (64.29%)	5 (35.71%)		2 (33.33%)	4 (66.67%)	
Boys	5 ((35.71%)	9(64.29%)		5 (62.50%)	3 (37.50%)	
Scoliosis, n (%)			0.430 ^c^			1.000 ^c^
Yes	8 (44.44%)	10 (55.56%)		4((44.44%)	5 (55.56%)	
No	6 (60.00%)	4 (40.00%)		3 (60.00%)	2 (40.00%)	
HFMSE_V0, median (IQR)	13 (5.25, 17)	9 (5, 21)	0.729 ^a^	48 (36.5, 57.5)	53 (48, 54.5)	0.798 ^a^
∆HFMSE, mean ± sd	6 ± 3.0884	1.2143 ± 2.8871	**< 0.01** ^**b**^	3 ± 2.582	4.2857 ± 5.4989	0.586 ^**b**^
SMN1, n (%)			1.000 ^c^			1.000 ^c^
0	13 (52.00%)	12 (48.00%)		5 (50.00%)	5 (50.00%)	
1	1 (33.33%)	2(66.67%)		2 (50.00%)	2 (50.00%)	
SMN2, n (%)			1.000 ^**c**^			0.462 ^c^
3	10 (47.62%)	11 (52.38%)		5 (41.67%)	7 (58.33%)	
2	4 (57.14%)	3 (42.86%)		1 (100%)	0 (0%)	
4	-	-		1 (100%)	0 (0%)	
CSF_LDH, median (IQR)	18 (14.25, 21.75)	16.5 (15, 21.75)	0.661^a^	18.43 ± 7.76	15 ± 4.04	0.320^b^
CSF_protein, median (IQR)	0.245 (0.23, 0.30)	0.225 (0.20, 0.31)	0.369^a^	0.30 ± 0.12	0.26 ± 0.09	0.554^b^
CSF_glucose, mean ± SD	3.50 ± 0.25	3.48 ± 0.29	0.885^b^	3.42 (3.31, 3.55)	3.36 (3.22, 3.50)	0.654^a^
CSF_chloride, mean ± SD	127.66 ± 2.55	127.61 ± 2.43	0.958^b^	128.10 ± 1.01	127.74 ± 2.17	0.703^b^

II: Spinal muscular atrophy type II III: Type III spinal muscle atrophy. *HFMSE* Hammersmith Functional Motor Scale, *CSF* cerebrospinal fluid, *IQR* interquartile range, *SD* standard deviation, *LDH* lactate dehydrogenase. a: Mann–Whitney rank test. b: *t*-test. c:Fisher's exact test

### CSF metabolic profiling of SMA

**Fig. 2:**
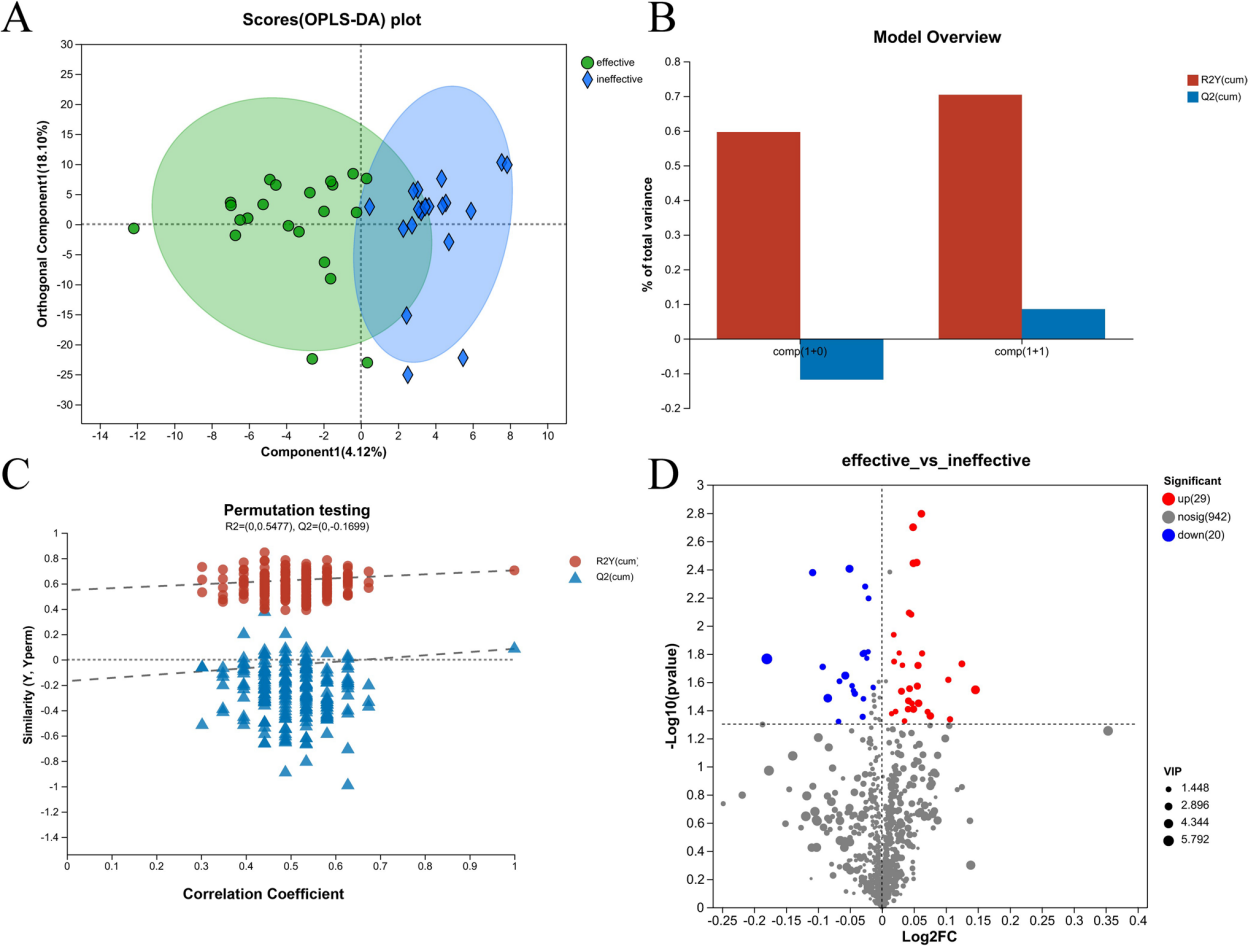
Orthogonal partial least squares discriminant analysis (OPLS-DA) scores scatter plot and volcano plot of the spinal muscular atrophy (SMA) with treatment effective and treatment ineffective. **A** OPLS-DA scores plot. **B** OPLS-DA principal components. **C** OPLS-DA model validation. **D** Volcano plot

To identify the CSF metabolites that can potentially predict the efficacy of SMA, we analyzed the CSF metabolites from patients with SMA types II and III, grouped according to treatment efficacy. However, the OPLS-DA analysis indicated that sample differentiation was not significant. In contrast, the differential analysis indicated that 30 CSF metabolites were significantly differentially expressed between the effective- and ineffective-treatment groups (Fig. 2, Table S1).

**Fig. 3:**
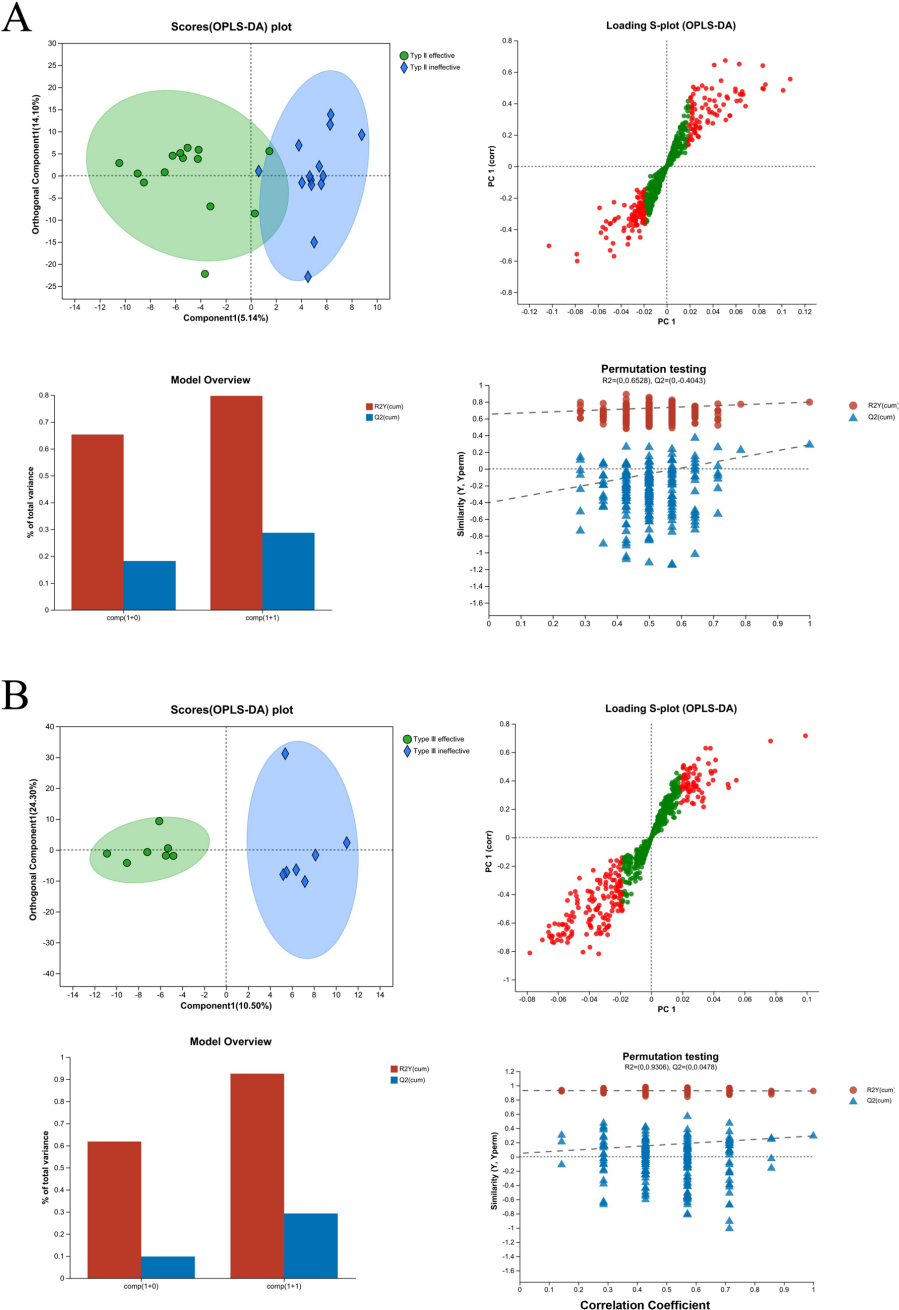
Orthogonal partial least squares discriminant analysis (OPLS-DA) scores scatter plot of the different type of spinal muscular atrophy (SMA) with treatment effective and treatment ineffective. **A** OPLS-DA scores scatter plot of SMA type II. **B** OPLS-DA scores scatter plot of SMA type III

Moreover, stratified OPLS-DA analyses indicated that the data were comparable between the effective- and ineffective-treatment groups for patients with SMA type II and III (Fig. 3).

**Fig. 4:**
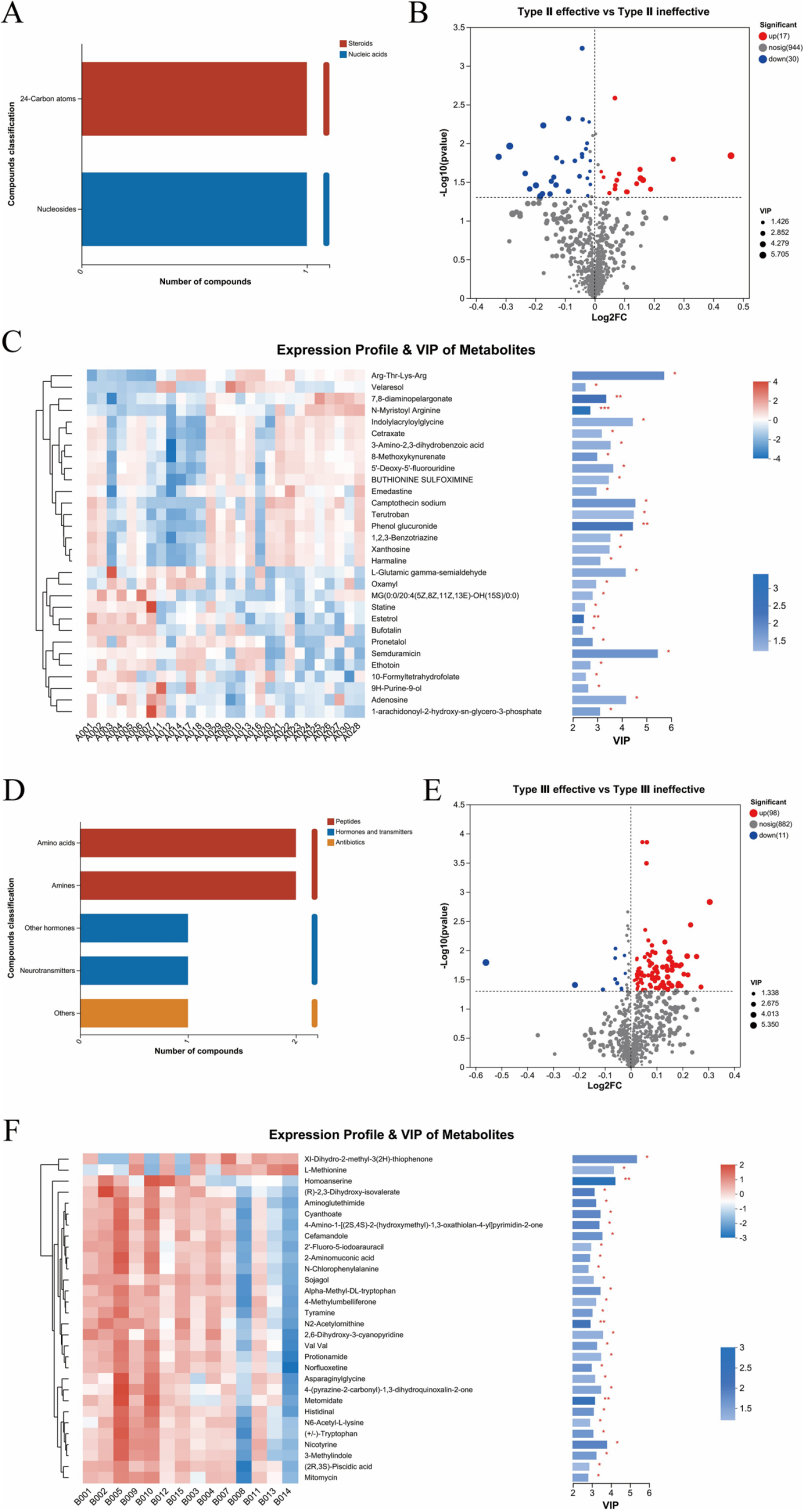
Different metabolites in the cerebrospinal fluid (CSF) of spinal muscular atrophy (SMA) type II and SMA type III with effective treatment and ineffective treatment. **A** Classes of metabolites in CSF of SMA type II. **B** Volcano plot of metabolites in the CSF of SMA type II. **C** Top 30 metabolites with differences based on the comprehensive judgment of *p* value and VIP of SMA type II. **D** Classes of metabolites in CSF of SMA type III. **E** Volcano plot of CSF metabolites SMA type III. **F** Top 30 metabolites with differences based on the comprehensive judgment of *p* value and VIP of SMA type III

For SMA type II, we identified two classes of CSF metabolites: steroids and nucleic acids (Fig. 4A). We identified 47 metabolites with *p* < 0.05, FDR < 0.2, and VIP > 1, according to the OPLS-DA model (Fig. 4B, Table S1). The top 30 metabolites with differences based on the comprehensive judgment of the *p* value and VIP are shown in Fig. 4C. For SMA type III, we identified three classes of metabolites in the CSF: peptides, hormones, and transmitters (Fig. 4D). A total of 109 metabolites with *p* < 0.05, FDR < 0.2, and VIP > 1, according to the OPLS-DA model, were identified (Fig. 4E, Table S2). The top 30 metabolites with differences based on the comprehensive judgment of the *p* value and VIP are shown in Fig. 4F.

### Enrichment analysis of CSF metabolomics

**Fig. 5:**
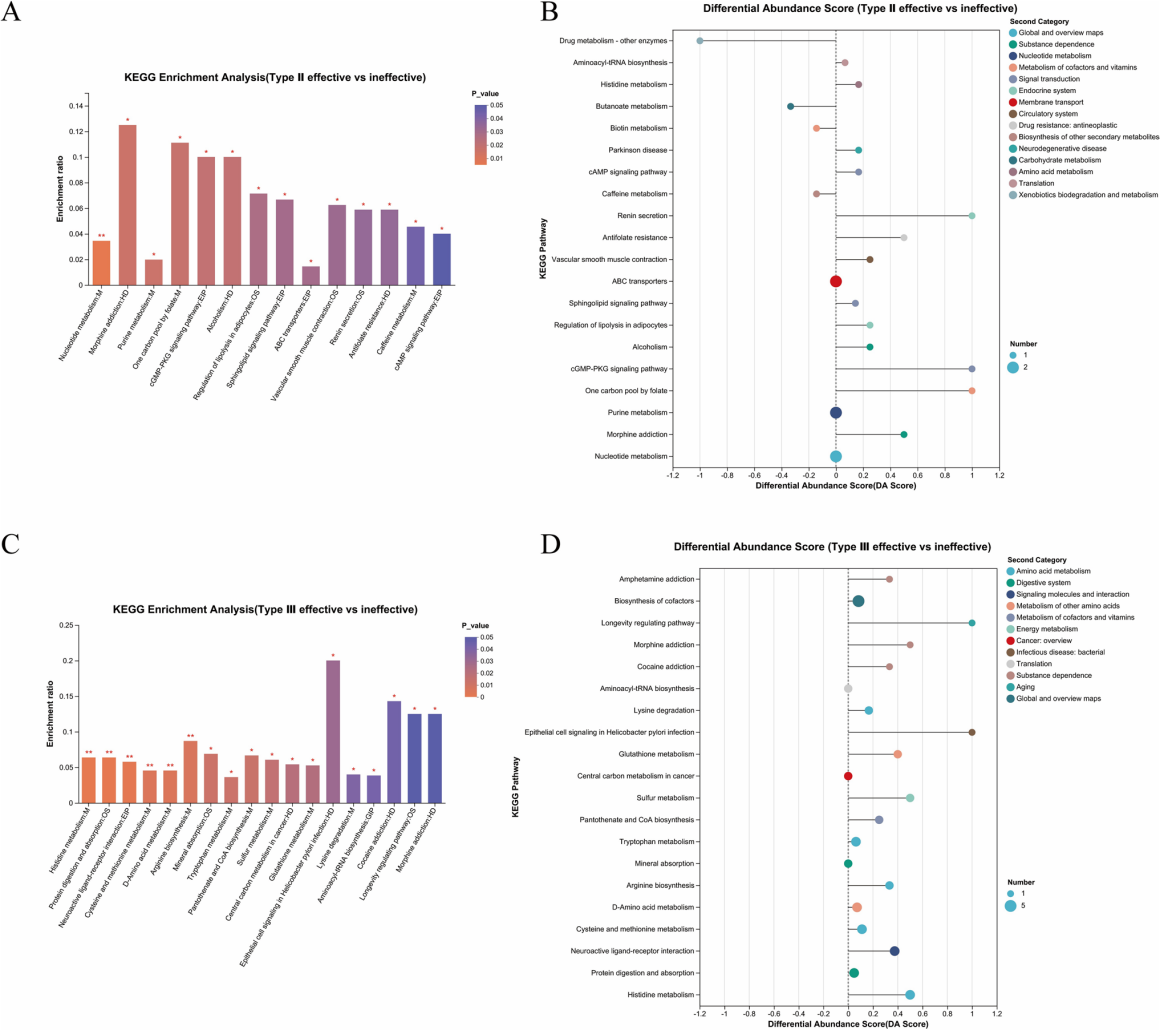
Enrichment analysis of cerebrospinal fluid (CSF) metabolomics. **A** Enrichment analysis of CSF metabolomics of SMA type III based on p value. **B** Enrichment analysis of CSE metabolomics of SMA type II based on abundance score. **C** Enrichment analysis of CSF metabolomics of SMA type III based on *p* value. **D** Enrichment analysis of CSE metabolomics of SMA type III based on abundance score. * *p* value or FDR < 0.05, **p value or FDR < 0.01, ***p value or FDR < 0.001

We performed KEGG enrichment analysis based on the results of the differential analysis. For SMA type II, KEGG enrichment analysis showed that CSF metabolites were primarily involved in including endocrine, biological signaling, and amino acid, vitamin, and nucleotide-purine metabolism. As for SMA type III, KEGG enrichment analysis showed that CSF metabolites were primarily involved in amino acid and purine metabolism as well as phospholipid metabolism (Fig. 5, Table S3).

### Correlation between CSF metabolites and clinical features

**Fig. 6:**
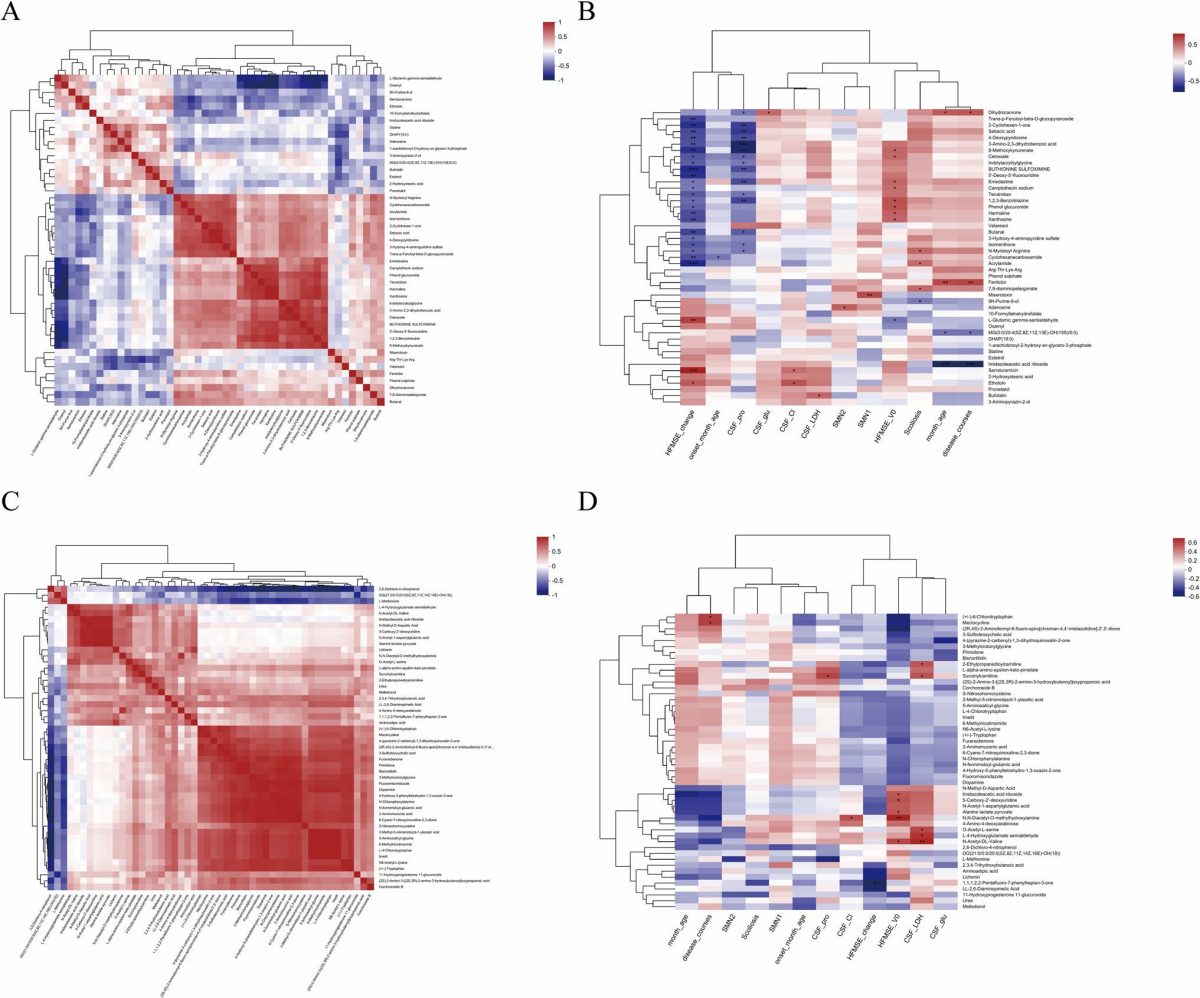
Correlation between cerebrospinal fluid (CSF) metabolites and clinical features. **A** Correlation between CSF metabolites of SMA type II. **B** Correlation between CSF metabolites and clinical features of SMA type II. **C** Correlation between CSF metabolites of SMA type III. **D** Correlation between CSF metabolites and clinical features of SMA type III. *SMN1*: Survival of Motor Neuron 1. *SMN2*: Survival of Motor Neuron 2. HFMSE: Hammersmith Functional Motor Scale Expanded. CSF: cerebrospinal fluid. LDH: lumbar disc herniation

We performed correlation analyses to further clarify whether the metabolites could be used as diagnostic markers to distinguish SMA treatment efficacy. The correlation between CSF metabolites is shown in Fig. 6A and C. The correlations between CSF metabolites and clinical features are shown in Fig. 6B and D. Notably, N-myristoyl arginine was negatively correlated with HFMSE change, while L-glutamic gamma-semialdehyde was positively correlated with HFMSE change (*p* < 0.05) in SMA type II. 1,1,1,2,2-Pentafluoro-7-phenylheptan-3-one was negatively correlated with HFMSE changes in SMA type III.

### Receiver operating characteristic curve (ROC) analysis

**Fig. 7:**
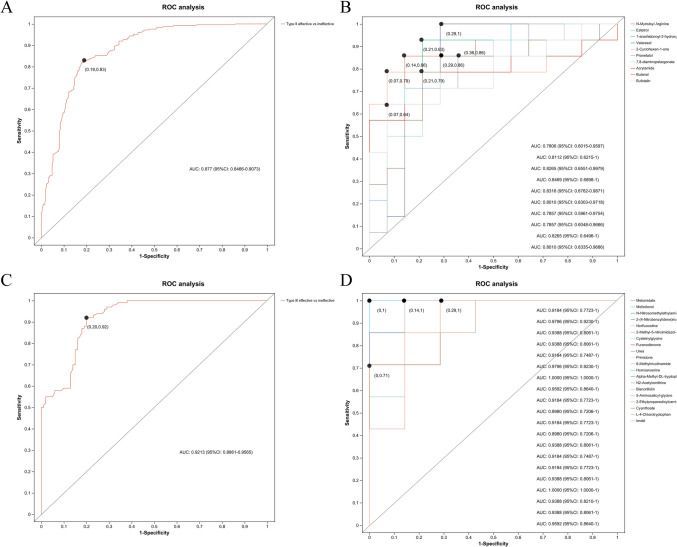
Receiver operating characteristic curve (ROC) analysis. **A** Multivariate ROC curve analysis of spinal muscular atrophy (SMA) type II, demonstrating the efficacy of identified metabolites in predicting treatment response, with an area under curve (AUC) value of 0.877 (95% CI: 0.8466–0.9073). **B** Univariate ROC curve analyses of SMA type II, highlighting the individual predictive performance of specific metabolites. **C** Multivariate ROC curve analysis of SMA type III, indicating a higher predictive accuracy with an AUC value of 0.9213 (95% CI: 0.8861–0.9565). **D** Univariate ROC curve analyses of SMA type III, outlining the effectiveness of selected metabolites in distinguishing treatment efficacy in this patient group. ROC: receiver operating characteristic curve. AUC: area under the curve

To further evaluate the prediction performance of the identified metabolites for treatment efficacy, we performed univariate and multivariate ROC curve analyses to distinguish between children with SMA type II and SMA type III. We first conducted a multivariate ROC curve analysis based on all previously identified differential metabolites with an area under curve (AUC) value of 0.877 (95% CI: 0.8466–0.9073, Fig. 7A) and an AUC value of 0.9213 (95% CI: 0.8861–0.9565, Fig. 7C), suggesting that differential metabolites had a certain accuracy in predicting SMA treatment efficacy (Fig. 7B, D, Table S1).

## Discussion

Previous predictions regarding nusinersen efficacy were based on analyses of the clinical characteristics of patients with SMA. Here, the common characteristics of patients in the effective-treatment group were young age at treatment initiation and short disease duration, which were significantly different between the two treatment groups. However, the predictive value of age and disease duration for nusinersen efficacy was poor in logistic regression analysis. Additionally, although *SMN2* copy number does not predict nusinersen efficacy in patients with type II or III SMA, it has been associated with disease severity prediction [19]. Clinical features that predict treatment efficacy are inconsistent in the current literature. Coratti et al. [20] showed that age at the start of treatment and the Motor Assessment Scale score at baseline predicted the effectiveness of nusinersen, but the *SMN2* copy number did not. In contrast, Osredkar et al. [21] analyzed factors predictive of efficacy in 61 patients with types I, II, and III SMA and found that efficacy was related to the *SMN2* copy number. However, Kotulska et al. [22] suggested that the improvement in HFMSE scores after nusinersen treatment was not related to the number of *SMN2* copies or to the age at which treatment was initiated. These findings indicate that predicting nusinersen efficacy based on clinical characteristics alone is unreliable. Necessitating a search for other promising predictors.

Here, CSF samples were collected from 42 patients with SMA at baseline. This study aimed to identify objective CSF metabolite biomarkers that predict treatment response. CSF metabolites did not separate well when grouped for efficacy without considering clinical typing factors, suggesting that the predictive value of CSF metabolites for treatment efficacy must be based on clinical typing. Furthermore, in a study by Errico et al. [23], no significant changes in CSF metabolomics were observed in the SMA cohort during the nusinersen loading and maintenance phases or the baseline period; however, differences in CSF metabolites between the treatment periods were observed after clinical typing. This may be because CSF metabolites are specific to clinical symptom severity. Therefore, we performed CSF metabolite analysis to predict clinical efficacy of nusinersen treatment in patients with type II and type III SMA.

By analyzing different pathways, we found that effective treatment of type II patients exhibited differences in several pathways, including endocrine, biological signaling, and amino acid, vitamin, and nucleotide-purine metabolism. SMN is implicated in nucleotide metabolism, such as the uridine-rich spliceosome and RNA synthesis [24, 25]. Additionally, SMN is a key protein involved in the assembly of spliceosomal small nuclear ribonucleoproteins (snRNP) in several cell types [26]. In the absence of SMN, there is a defect in the U12 spliceosome that leads to abnormal splicing of the gene. SMN also contributes to the assembly of mRNA-binding protein complexes, such as neuronal distal messenger ribonucleoprotein transport particles (mRNPs), which facilitate mRNA transport [27]. In this study, more metabolites were involved in nucleotide metabolism in the effective-treatment group for patients with type II SMA.

The differential metabolic pathways of effective-treatment patients with type III focus on amino acid and purine metabolism as well as phospholipid metabolism. Martina Zandl-Lang et al. found dysregulation of lipid metabolism in SMA patients, which was partially reversed by nusinersen [28]. They found that levels of amines associated with lipid metabolism, such as sphingosine, myristoyl ethanolamine, and palmitoyl ethanolamine, were also elevated in the patients by at least approximately 1.9, as compared with controls, which was generally consistent with our study. Francesco Errico et al. found that in patients with type III SMA, nusinersen mainly regulated amino acid metabolism [23]. Therefore, the enrichment of metabolites in the mentioned metabolic pathways at baseline may have a synergistic effect on nusinersen treatment, ultimately resulting in a favorable therapeutic outcome.

The present study found significant differences in the CSF metabolites, 1-arachidonoyl-2-hydroxy-sn-glycero-3-phosphate, N-myristoyl arginine, and 7,8-diaminopelargonate between the effective and ineffective nusinersen treatment groups from patients with type II SMA.

1-arachidonoyl-2-hydroxy-sn-glycero-3-phosphate is a lysophosphatidic acid (LPA) with a wide range of biological activities. The release of purines from microglia activates LPA, which can lead to demyelinating spinal cord injury (SCI) [29]. In a mouse nerve injury model, a dramatic increase in LPA was observed in the dorsal horn of the spinal cord [30]. In a genetic model of amyotrophic lateral sclerosis (ALS) in mice [31], LPA expression was upregulated in the sciatic nerve and skeletal muscle. A deficiency in LPA receptors delays the onset of the disease and slows neurological decline in ALS mice. In our study, 1-arachidonoyl-2-hydroxy-sn-glycero-3-phosphate was found to be negatively related to the efficacy of nusinersen, and its ability to reverse predict the efficacy of nusinersen was confirmed.

Recent studies have found that neuroinflammation plays an important role in the pathogenesis of SMA and is closely related to the development and severity of SMA. Maruša Barbo et al. [32] found that the polymorphisms of inflammation-related genes were associated with SMA susceptibility, disease type, age of symptom onset, and motor and respiratory function, indicating that neuroinflammation may affect the development of SMA. Qiang Zhang et al. [33] found that after 6 months of treatment with nusinersen, Eotaxin and MIP-1β levels in the CSF were significantly decreased, while IL-2, IL-4, and VEGF-A levels were increased in SMA patients. Although nusinersen can increase the level of SMN protein and improve the clinical symptoms of some patients, it cannot completely cure SMA. Neuroinflammation may affect the therapeutic effect of nusinersen, and residual neuroinflammation may become a new therapeutic target [34]. In the study, we found that 1,1,1,2,2-pentafluoro-7-phenylheptan-3-one and N-myristoyl arginine were significantly and negatively correlated with HFMSE change. They are both associated with neuroinflammation [35–37].

1,1,1,2,2-Pentafluoro-7-phenylheptan-3-one, also known as FKGK11, is a selective inhibitor of the group VIA calcium-independent phospholipase A2 (GVIA iPLA2) [38, 39]. GVIA iPLA2 is ubiquitous in different brain regions, such as the hypothalamus, hippocampus, cerebral cortex, midbrain, and striatum [40]. Various neurodegenerative disorders are believed to be associated with deficiencies in GVIA iPLA2 expression [41], including infantile neural axonal dystrophy, atypical neural axonal dystrophy, and adult-onset dystonia Parkinson's disease. Previous studies on iPLA2 have shown that it is the major phospholipase responsible for the release of docosahexaenoic acid (DHA) from glycerophospholipids [42]. iPLA2β knockout mice have minimal neuropathological changes at birth but show signs of dyskinesia, cerebellar neuron loss, and striatal α-synuclein accumulation as they age. In addition, aged IPLA2β knockout mice also showed activation of microglia and astrocytes, as well as increased tumor necrosis factor α (TNFα) production, suggesting a role for iPLA2 in mediating inflammatory responses with aging [43]. In the present study, 1,1,1,2,2-Pentafluoro-7-phenylheptan-3-one expression was reduced in the effective treatment group of patients with type III disease. Additionally, it was significantly negatively correlated with the HFMSE scores. This demonstrates the predictive value of 1,1,1,2,2-Pentafluoro-7-phenylheptan-3-one for nusinersen efficacy. This also suggests that GVIA iPLA2 may play a synergistic role in the treatment of SMA with nusinersen. However, research on this topic is lacking.

Some limitations exited in the present study. Although we identified CSF metabolites as promising predictors of nusinersen treatment efficacy, further validation of their predictive value with larger sample sizes from multiple medical centers is required. Additionally, while some differential metabolites have plausible explanations for their utility, others lack such explanations owing to limited research. These are possible directions for future research. To ensure an objective and uniform evaluation of treatment efficacy, the HFMSE was used to assess motor function. However, this scale may not accurately evaluate changes in motor function in type III patients with mild symptoms owing to the ceiling effect. The kind of patients may score high based on the scale and may reached the highest score after therapy, causing a condition that motor function is better than that reflected by the score. Seven of the 21 patients who did not respond had an improvement of less than 3 points on the HFMSE score. But there have been improvements in other areas. For example, a 7-year-old boy with type II could only move his fingers slightly and swallow liquid food before treatment. After treatment, he could operate a wheelchair and chew solid food, and the number of hospitalizations due to infection per year has decreased significantly. This could not be reflected in the HFMSE score. Patients with comorbidities were excluded and may lead to biases for that comorbidities may affect therapeutic effect, which might limit the generalizability.

## Conclusions

This study identified CSF metabolites that are predictive of nusinersen efficacy. These metabolites include 1-acryloyl-2-hydroxy-sn-glycero-3-phosphate, N-myristoyl arginine and 1,1,1,2,2-pentafluoro-7-phenylheptan-3-one. The findings of this study may aid in the discovery of new adjunctive therapies that enhance the efficacy of nusinersen therapy.

## Supplementary information

ESM 1 Table S1 Differentially Expressed CSF Metabolites in SMA Types II and III Based on Treatment Efficacy (XLSX 16 kb)

ESM 2 Table S2 Comprehensive List of CSF Metabolites Identified in SMA Type III with Statistical Significance (XLSX 103 kb)

ESM 3 Table S3 KEGG Pathway Enrichment Analysis of Differentially Expressed CSF Metabolites in SMA Types II and III (XLSX 16 kb)

## Data Availability

The datasets generated during and/or analysed during the current study are not publicly available but are available from the corresponding author on reasonable request.
